# Chronic household air pollution and exposure patterns among Himalayan nomads

**DOI:** 10.1038/s41370-024-00656-z

**Published:** 2024-03-05

**Authors:** Catlin I. Powers, Linyan Li, Majid Ezzati, James P. Butler, Corwin M. Zigler, John D. Spengler

**Affiliations:** 1 Department of Environmental Health, Harvard T.H. Chan School of Public Health, Boston, MA, USA; 2 School of Data Science, City University of Hong Kong, HKSAR, China; 3 Department of Infectious Disease and Public Health, City University of Hong Kong, HKSAR, China; 4 MRC Centre for Environment and Health, School of Public Health, Imperial College, London, UK; 5 Department of Medicine, Harvard Medical School, Boston, MA, USA; 6 Departments of Statistics and Data Sciences, University of Texas at Austin and Dell Medical School, Austin, TX, USA

**Keywords:** Household air pollution (HAP), Himalayan nomads, Carbon monoxide (CO), Personal exposure, Biomarker, Solid fuels

## Abstract

**Background:**

Household air pollution (HAP) is a major risk factor of non-communicable diseases, causing millions of premature deaths each year in developing nations. Populations living at high altitudes are particularly vulnerable to HAP and associated health outcomes.

**Objectives:**

This study aims to explore the relationships between activity patterns, HAP, and an HAP biomarker among 100 Himalayan nomadic households during both cooking and heating-only periods.

**Methods:**

Household CO was monitored in 100 rural homes in Qinghai, China, at 3500 m on the Himalayan Plateau among Himalayan nomads. Carboxyhemoglobin (COHb) was used as a biomarker to assess exposure among 100 male and 100 female heads of household. Linear mixed-effects models were used to explore the relationship between COHb and activity patterns.

**Results:**

Cooking periods were associated with 7 times higher household CO concentrations compared with heating periods (94 ± 56 ppm and 13 ± 11 ppm, respectively). Over the three-day biomarker-monitoring period in each house, 99% of subjects had at least one COHb measurement exceeding the WHO safety level of 2%. Cooking was associated with a 32% increase in COHb (*p* < 0.001).

**Impact Statement:**

This study on household air pollution (HAP) in high-altitude regions provides important insights into the exposure patterns of nomadic households in Qinghai, China. The study found that cooking is the primary factor influencing acute carbon monoxide (CO) exposure among women, while heating alone is sufficient to elevate CO exposure above WHO guidelines. The results suggest that cooking-only interventions have the potential to reduce HAP exposure among women, but solutions for both cooking and heating may be required to reduce COHb to below WHO guidelines. This study’s findings may inform future interventions for fuel and stove selection to reduce HAP and exposure among other populations.

## Introduction

According to the World Health Organization (WHO), 3.8 million premature deaths each year are attributable to household air pollution (HAP) based on the role of HAP as a major risk factor for the most prominent non-communicable diseases in developing nations: pneumonia, ischemic heart disease, stroke, chronic obstructive pulmonary disease (COPD) and lung cancer [1, 2]. One of the primary components of HAP that has been associated with adverse health outcomes is carbon monoxide (CO). CO is a toxic gas that inhibits energy production in cells and affects heart, brain, and muscular functions [3].

With approximately 940 million people cooking and heating with polluting solid fuels (dung, wood, crop residues, and coal), China is estimated to have one of the largest burdens of disease from HAP [4, 5], especially in the rural areas where HAP can be a predominant source of exposure to air pollutants [6, 7]. Lack of personal exposure and activity data from larger studies, combined with diverse stove-fuel combinations, has made it difficult to assess the health implications of pollution levels measured in Chinese homes [8].

Household levels of CO_2_ were assessed in a study involving 14,928 households across eighty-eight Chinese cities, resulting in spatial patterns of CO_2_ emissions ranging from 0.39 to 1.17 t CO_2_/ person [9]. However, fewer studies have been carried out on the Himalayan Plateau, which covers 40% of China [10]. In previous studies in this region, Kang et al. showed warning levels of total suspended particles and trace metals in Tibetan tents during cooking with yak dung, exceeding not only the indoor air quality guidelines set by the World Health Organization but also surpassing the outdoor air levels in the Nam Co area by more than 10^4^–10^6^ times [11]. Gao et al. compared household PM_2.5_ in homes using solid biomass versus methane [12]; Li et al. monitored activity patterns and personal CO among nine women [13]; Carter et al. measured PM_2.5_ levels in 204 households in eastern Tibetan Plateau and found concentrations in winter over two times higher than in summer [14]; Ni et al. measured both indoor and personal CO in a study of 205 women cooking with biomass in Sichuan, China [15]. Ye et al. conducted a comprehensive HAP exposure assessment for 24 households in the eastern Tibetan Plateau and showed that substantial HAP exposure was prevalent. Immediate actions need to be taken to mitigate its associated potential negative health outcomes [16]. Sclar and Saikawa took 28 measurements of PM_2.5_ and black carbon (BC) and found high HAP concentrations for residents in Tibet [17]. To the best of our knowledge, no studies in this region of China reported biomarkers of CO exposure, which poses limitations on the development of effective interventions and the implementation of policy changes.

Investigating the health risks of this population living at high altitude presents an important public health topic, as there are more than 140 million high-altitude residents living at >3000 meters above sea level around the world [18]. The low partial pressure of oxygen at these altitudes is known to increase production of particles of incomplete combustion (PICs) during combustion of fuels for heating and cooking [10]. Further, lower competition from oxygen at hemoglobin binding sites may exacerbate the effects of CO and other constituents of HAP along with their associated health outcomes [3]. Evidence suggests that Himalayans may have genetic adaptations that compensate for lower atmospheric partial pressure of oxygen through alterations in respiratory physiology [19]. The effect of these changes on CO uptake and offloading, however, is unknown.

In this study, we aim to characterize high altitude HAP exposures among Himalayan nomads by monitoring household CO in 100 rural homes in Qinghai, China at 3500 m on the Himalayan Plateau, and using carboxyhemoglobin (COHb) as a biomarker to assess exposure among 100 male and 100 female heads of household. Our study population had similar housing structures, gender specific roles and all heated and cooked with dried yak dung, providing a unique opportunity for us to isolate the relationships between activity patterns, HAP, and COHb and to gain insight into the role of heating versus cooking exposures. In addition, we hypothesize that cooking would be the predominant factor influencing acute CO exposures among women but that heating alone would be sufficient to elevate CO exposures above WHO guidelines. To our knowledge, our study is among the largest evaluation of HAP exposure using the partial pressure of exhaled CO (pCO) and COHb as a biomarker. This study community remains nomadic. For the nomadic populations, conditions are similar or more challenging due to land degradation and climate change. Yak dung is still the primary fuel, and many have gone back to adobe stoves exclusively due to supply chain issues. Our study will provide insight into the understudied exposure patterns in the nomad households in high-altitude regions, and our results may be helpful for future interventions involving the fuel and stove for cooking and heating to reduce HAP and exposures among other populations.

## Methods

### Study area and participants

Our study area, Jianzha County in Qinghai province, China (35°52’N 101°51’E, altitude: 3500 m), is located in the Himalayas where heating is an important energy requirement throughout the year. The primary livelihood of study participants was herding, and participants migrated multiple times per year at altitudes up to 5400 m. Among our study population, income was earned primarily from selling animal products, engaging in seasonal labor, and collecting local medicinal herbs, and was below the international poverty line at $0.91 per person per day [20].

We discussed potential participation with all 147 households in the local community. An initial selection was conducted based on whether or not the heads of household were both present during our initial visit to provide informed consent. Among these households, 100 households were randomly selected in this study. All study protocols were reviewed and approved by the Harvard T.H. Chan School of Public Health Institutional Review Board and Chinese Ministry of Science and Technology.

### Household air pollution sampling

CO monitors ran continuously and simultaneously for 44 sampling days in all 100 participating households from March to April 2012, yielding a total of 4400 household-days. Samples were logged at 5-min intervals. Monitors were located approximately one meter from the stove and one meter above the ground—near breathing level for people sitting around the stove. The sensors employed were NAP-505 electrochemical CO gas sensors (Range: 0–1000 ppm; Accuracy: ±2%; Nemoto, Burgermeester Haspelslaan 53, 1181NB Amstelveen, The Netherlands) embedded in EL-USB-CO Data Loggers (Lascar Electronics, Module House, Whiteparish, Salisbury, SP5 2SJ, United Kingdom). Duplicate measurements were conducted in 2% of homes. Background CO levels were measured outdoors and found to be below the limit of detection. Outdoor temperature was monitored continuously outside the home of the lead field assistant throughout the study period. The outdoor daily average temperature ranged from −3 to 9 °C. The effects of temperature and pressure on sensor output were assessed at altitude and corrected for in the calculation of the CO concentrations.

For each 5-min of the day, boxplots of CO measurements from all 100 households across all 44 days were generated to illustrate diurnal variation in median and interquartile CO concentration. The average of CO when active cooking stopped was calculated to determine the contribution from combustion for heating.

### Subject activity and exposure assessment

Field assistants visited each study home three times per day (morning, afternoon, evening) on three consecutive days in March-April 2012. During these visits, field assistants recorded time-activity budgets of 100 male and 100 female heads of household and administered daily surveys characterizing smoking and other sources of personal exposure aside from the stove (incense, kerosene, candles, etc.). Each field assistant and each participant were provided a watch to facilitate detailed time recordings; time syncing was checked by field assistants before the first visit of each day. Field assistants recorded the present activity of the participant (cooking, herding, other outdoor, other indoor, resting, collecting water, or collecting fuel). Participants were asked for a summary of their activities since waking up (if a morning measurement) or since the last visit (if an afternoon measurement). Location of activity was described as indoors or outdoors, and time spent on each activity daily was reported by subjects. Subjects also reported number of cigarettes smoked during activity.

We measured CO in exhaled breath (pCO) for practical reasons of feasibility, cost and participant burden. Laboratory and field validation studies found pCO exhaled breath methods compared well to gold standard blood-gas methods with r-square correlation values between 0.88 and 0.98 [21–23]. We measured pCO with a ToxCO™ Breath CO Monitor (Range: 0–600 ppm, Accuracy: ±5%, Bedfont Scientific Ltd, Station Rd, Harrietsham, Maidstone, Kent ME17 1JA, UK). This instrument employs an electrochemical sensor to measure CO (reported in ppm) in exhaled breath and then calculates COHb using the Haldane equation and physiological parameters consistent with healthy males at sea level [24]. The Haldane equation is a well-established and validated approach to correlate exhaled CO and COHb levels, and since the participants in our study are fully acclimated to the altitude of their location, it is reasonable to assume the key parameters in the equation (e.g., blood O_2_ level and the ratio of the affinity of blood for CO to that for O_2_) used for the sea-level population can be also applied to our study population.

During each visit, assistants administered the ToxCO™ breath test. The test was conducted outdoors to avoid contamination from indoor CO. Participants were asked to inhale and hold their breath for 15 s before exhaling completely into the ToxCO™ mouthpiece, in accordance with ToxCO™ standard operating procedures. This was repeated three times and values were averaged. All breath monitors were calibrated weekly in the field using the calibration kit supplied by the manufacturer. Exposures during each activity were compared by averaging COHb (calculated from pCO) within activity and gender.

### Participant characteristics and activity patterns

A baseline survey was conducted to collect information on house types, stove types, smoking status, number of stoves, socio-economic status (SES) rank, number of family members, age of each child, estimated time spent collecting fuel, and smoking status. Specifically, SES ranking was conducted by the council of village elders. Each elder separately ranked all study households from 1 to 100 based on ownership of animals, land, and material items as well as on number of children, education level, and other factors influencing future opportunity. The elders then debated these rankings to arrive at a final ranking by consensus. Women were asked whether they carried children on their backs while cooking (always, usually, sometimes, seldom, never), whether they let their children play outside in winter (always, usually, sometimes, seldom, never), and whether they ask their children to assist with their father or mother.

### Statistical analyses

We fitted linear mixed-effects models with subject-specific random intercepts to investigate the relationship between COHb and activity patterns. COHb was log transformed to account for its positive skewness and regressed against self-reported activity type prior to COHb measurement (i.e., cooking, other outdoor, other indoor, and resting), mean 24-h CO concentration (ppm) in home, age, weight, sex, and smoking within 4 h prior to measurement. Contributions from incense, butter lamps, and other indoor combustion sources were analyzed based on observed and reported usage in religious ceremonies but found to be insignificant and excluded from the model. All statistical analyses were performed in R version 4.1.2.

## Results

Homes visited in this study were mostly one-room adobe houses with a large bed platform where family members slept and ate their meals. Families reported heating their homes throughout both the day and night, stoking the fire before bed and leaving yak dung fuel smoldering in the stove overnight to heat the bed platform.

Children between 2 and 7 years of age helped their mothers and played both indoors and outdoors. After the age of 7, male children accompanied and assisted their fathers while female children accompanied and assisted their mothers. Thirty percent of women reported that they tried to prevent their children from playing outdoors in winter (always or most of the time).

**Fig. 1:**
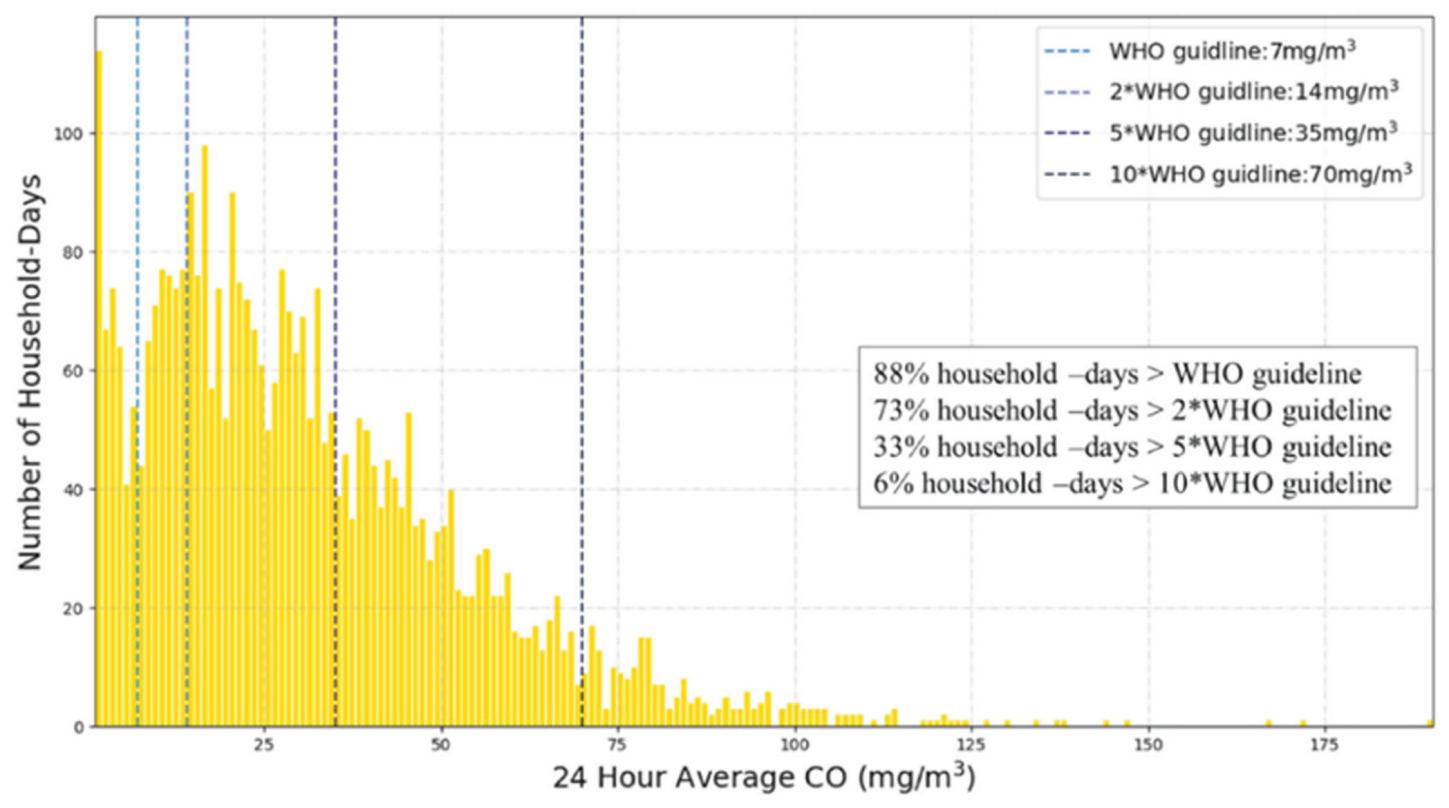
Distribution of household CO compared with WHO guidelines. 24-h average CO in homes exceeded WHO 8-h CO poisoning guideline of 9 ppm (vertical line) on 100% of household-days. CO was measured continuously in 100 homes, during 44 days, for a total of 4400 total measurement-days.

Of all 4400 household-days measured in this study, 88% had average 24-h indoor CO that exceeded WHO 24-h CO poisoning guidelines of 7 mg/m3 (6.1 ppm) (Fig. 1). Daily peak 15-min CO exceeded the WHO 15-min exposure ceiling of 87 ppm (99.6 mg/m^3^) in 73% of all 4400 household-days in our study.

**Fig. 2:**
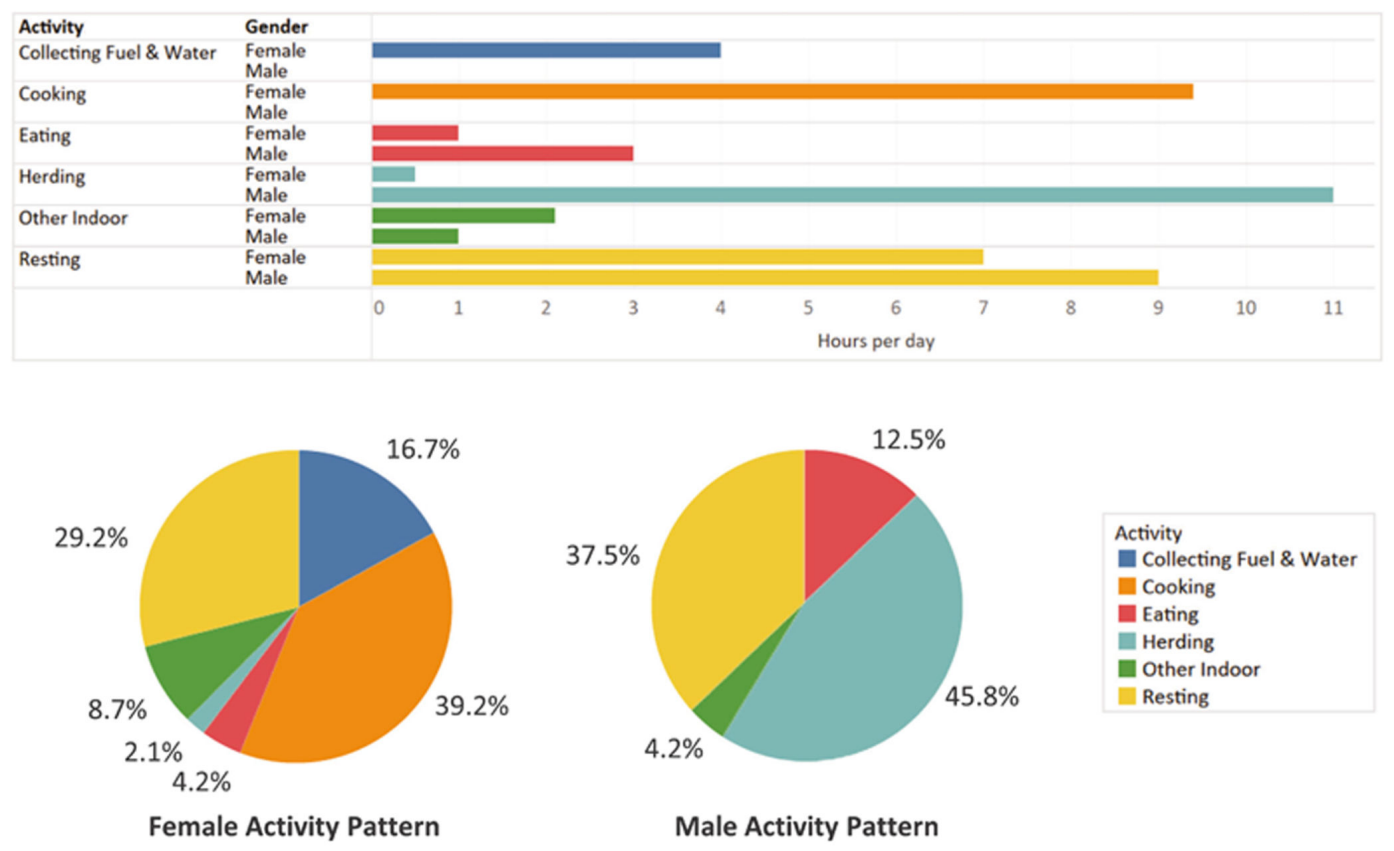
Male and female activity patterns over a 24-h day. On average, participants spent 8–10 h resting at nighttime. Men spent the majority of their daytime hours outdoors herding. Women spent the majority of their daytime hours cooking (9.4 h), collecting fuel (2.3 h) and collecting water (1.7 h).

**Fig. 3:**
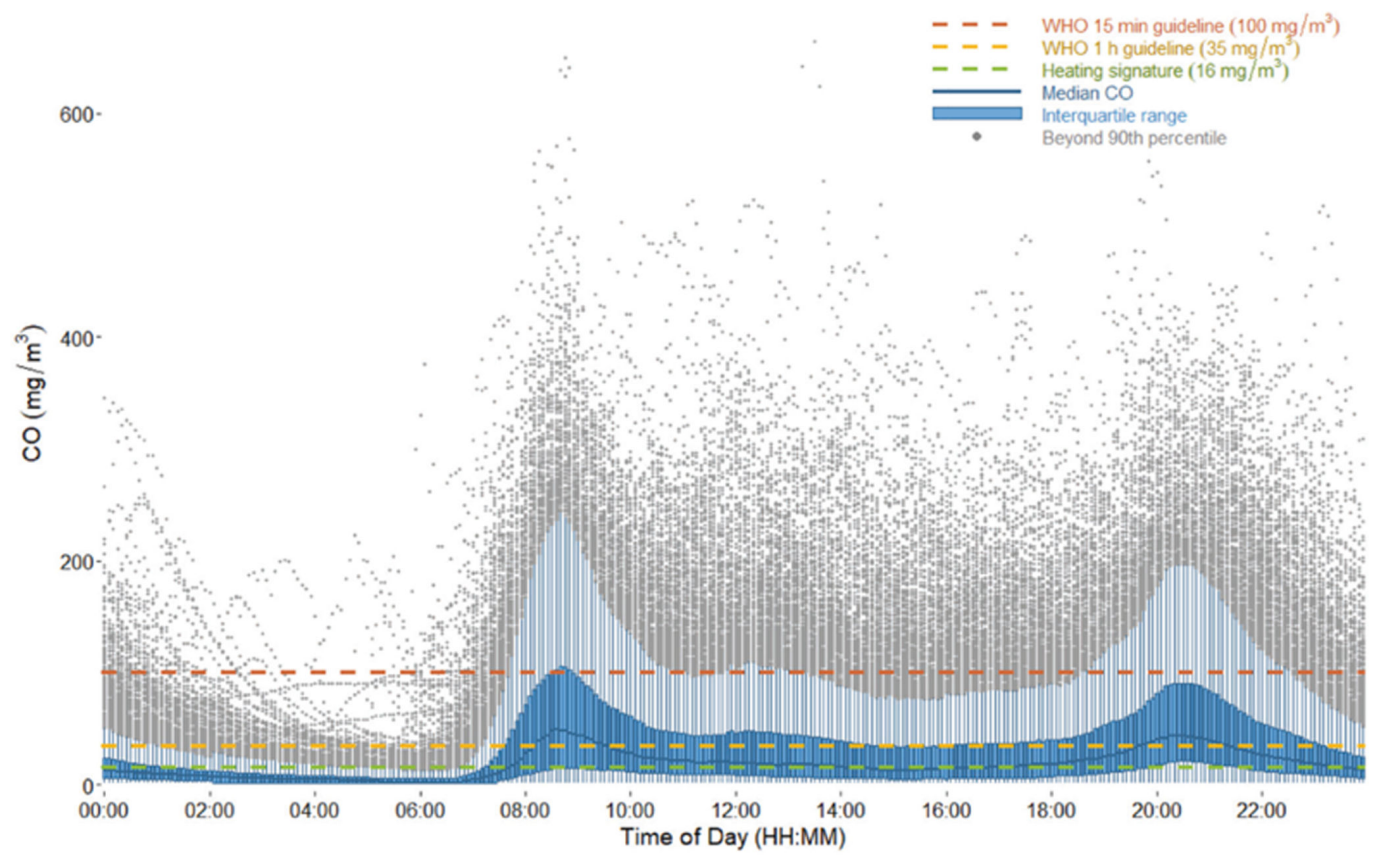
Diurnal variation in household CO across all household-days. Five-minute boxplots of continuous CO across all household-days show the emergence of a diurnal trend with peaks corresponding to meal times. Black line represents median. Green represents the interquartile range. Measurements beyond the 90th percentile of 5-minute measurements reached above 700 ppm CO but were excluded here in order to provide more detail in the lower measurement range. Median and interquartile range are skewed low due to imperfect overlap of cooking times in study homes. Red horizontal line represents average heating signature (13 ppm CO).

On average, males (age 7 and older) spent 46% of their time herding animals and 54% indoors (inclusive of sleeping) (Fig. 2). Women spent 81% of their time indoors, over 9 h of which were spent cooking (Fig. 2). As seen in Fig. 3, cooking periods typically occurred between 08:00 and 21:00, and this led to higher indoor CO levels during the day. The daytime average concentration was 94 ± 56 ppm (107.6 mg/m^3^) with an average daily maximum of 160 ± 102 ppm (183.2 mg/m^3^). Families spent 1–2 h per day having meals together on the bed platform. The nighttime average CO concentration was 13 ppm (14.9 mg/m^3^).

**Fig. 4:**
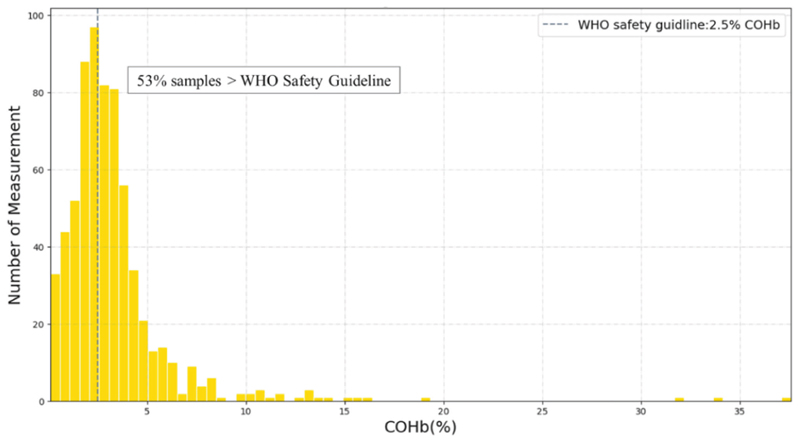
Distribution of COHb measurements compared with WHO guidelines. Mean COHb was 4.6 ± 3.9%. Min COHb was 0.4%, consistent with endogenous levels [25]. Max COHb was 37.6%, consistent with acute poisoning [3]. Vertical line represents the WHO CO guideline level of 2.5% COHb [31].

**Fig. 5:**
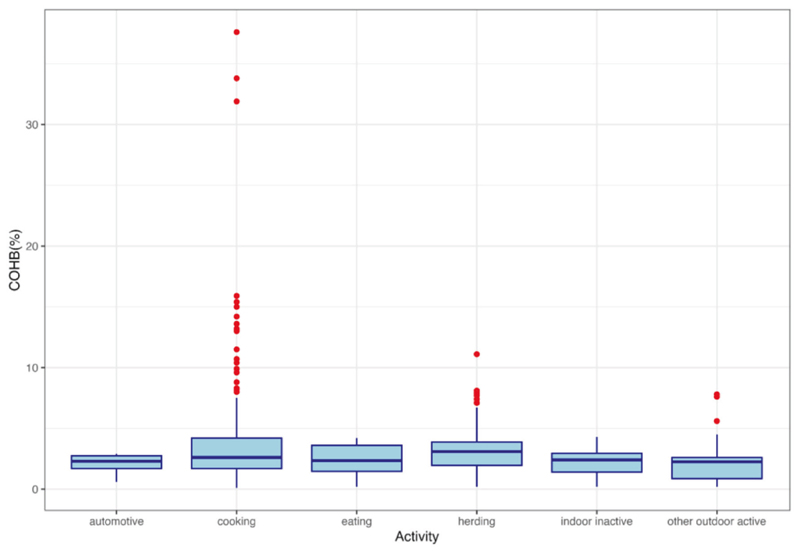
COHb level by activity patterns. Boxplots of the COHb measurements, grouped by activity patterns. Cooking has the largest number of outliers indicating alarmingly high COHb levels, followed by herding.

Among all 1800 COHb measurements taken on male and female heads of household during 600 measurement-days, 53% were above the WHO recommended COHb threshold (Fig. 4). Population mean COHb was 4.6 ± 3.9%. The lowest COHb measurement was 0.4% (consistent with endogenous production) and the highest measurement was 37.6% (consistent with acute carbon monoxide poisoning) [3, 25]. Based on the association between COHb and activity patterns shown in Fig. 5, it is evident that the level of COHb is notably higher during cooking and herding. The outliers demonstrate the presence of extreme COHb values during cooking, highlighting the cooking-related factors with elevated COHb levels.

COHb was above the 2% clinical threshold during 62% of observations among women and 74% of observations among men. However, COHb was above the 7% US OSHA occupational permissible limit during 11% of measurements among non-smoking women and only 2% of measurements among non-smoking men. Maximum COHb among women was 37.6% compared with 11.1% among men. 91% of COHb measurements >10% were recorded after cooking. On average, women spent about 9 h per day cooking. Linear mixed modeling indicated that cooking increased COHb by 32% after controlling for other factors.

Pearson’s correlations were significant between COHb and 24-h indoor CO (*r* = 0.43, *p* < 0.0001) and daily max CO (*r* = 0.45, *p* < 0.0001). Specifically, for females who spend most of their time indoors, the Pearson’s correlations between COHb and 24-h indoor CO (*r* = 0.51, *p* < 0.0001) and daily max CO (*r* = 0.54, *p* < 0.0001) were more substantial. The fraction of time each day that a subject’s home exceeded the average heating signature (13 ppm CO) was correlated with subjects’ COHb (*r* = 0.32, *p* < 0.0001).

According to the linear mixed-effects model, CO exposures were highest amongst women after cooking (COHb 4.2 ± 5.3%) and among men after herding, both for those who smoked during the herding activity (COHb 4.4 ± 4.9%) and for those who did not smoke four hours prior to the measurement (COHb 3.2 ± 1.6%) (Table 1). Cooking exposures among women were statistically similar to exposures among smoking men. Eating was associated with a significant elevation in COHb among both men (2.6 ± 1.5%, *p* < 0.0005) and women (2.3 ± 1.1%, *p* < 0.05) compared with resting levels. After controlling for key variables using mixed effects modeling, cooking increased COHb by 32% (β_1_ = 0.281, *p* < 0.001, Model 1) and was the only statistically significant driver of COHb apart from smoking.

## Discussion

In this study, we characterized exposure patterns to HAP in a nomads community in Qinghai, China by recording activity patterns, measuring 24-hour household CO levels, and calculating COHb concentrations based on CO measured in exhaled breath. The majority of our CO and COHb measurements exceeded the WHO safety guidelines, indicating that the nomad families were exposed to severe indoor air pollution primarily from using the traditional stoves.

Our research indicated that, in the cold climate and high altitude of Qinghai, cooking dominated the CO exposure among women. Women were predominantly the cook in the nomad families, and they had significantly higher CO exposure during cooking time than during the rest of the day. Cooking periods CO levels (94 ± 56 ppm) were associated with 7 times higher house-hold CO concentrations compared with overnight heating periods (13 ± 11 ppm). On the other hand, heating and smoking dominated CO exposure among men. Both men and women were exposed to elevated CO during mealtimes for 1–2 h/day, and to the background heating-signature overnight for 8–10 h. These risks were also significant to children in this population, given their activity patterns provided by their parents.

In addition to cooking and heating, our study population were exposed to CO from two other sources: smoking and leakage of exhaust fumes. Cigarette smoking was viewed as a way of combatting dizziness upon rapid altitude ascent during herding, as well as being an important social activity among men. Smoking was associated with elevated COHb (4.4 ± 4.9%) and even men who did not report smoking during activity exhibited elevated COHb (3.2 ± 1.6%) when they were outdoors. COHb among men reporting that they did not smoke within four hours immediately before the measurement was higher than expected, which indicated that the COHb level might not be determined by short-term behavior, but is the result of cumulated exposure. Since the data collected only reflected smoking behavior during the four hours prior to the measurement, further data collection is needed to fully explain this phenomenon. On the other hand, there is limited potential for exposure from to motorcycle exhaust or chimney-smoke, since men spend about half their time herding and travel on foot, typically far from homes and roads. Moreover, both women and men were exposed to HAP while sleeping. Their bed platform was heated underneath with dung fuel and exhaust often leaked out over the bed. At the beginning of the day, 33% of participants showed COHb levels which exceeded the WHO CO guideline. This concurred with nighttime household CO levels, which suggested smoke leakage from the kang-stoves usually maintaining a smoldering fire through the night.

Our measurements of CO in homes and COHb among men and women were consistent with other studies of air pollution and the use of biomass fuels, including Raiyani et al. in India [26], Torres-Dosal et al. in Mexico [27], and Zhou et al. in China [28]. Taken together, these studies clearly indicated that these populations were at risk of adverse health outcomes associated with CO exposure.

It was not feasible to directly measure COHb by obtaining blood samples. Given the much lower barometric pressure of oxygen at the study altitude, COHb calculated from pCO using the Haldane equation is likely to underestimate true COHb levels due to the assumption of negligible reduced hemoglobin (Hb), which does not hold true in low-oxygen, high-altitude environments [29]. Due to field-conditions, this study calculated COHb based on pCO measurements and not blood-gas techniques. Although not the gold standard, this method has been validated by several laboratory and field studies with a high degree of concurrence with blood-gas measurements. In addition, field studies have demonstrated that exhaled carbon monoxide markers, may be used as a biomarker of recent HAP exposures [30]. The COHb measurements obtained in our study provided insights on behavioral factors, gender differences and contribution sources. Further, COHb levels in this populations enabled comparisons to clinical studies associating COHb levels with COPD, pneumonia, respiratory infection, cognitive deficits, increased cardiac anomalies, and all-cause mortality [3].

Continuous CO monitoring in off-grid settings presents several challenges. HOBO CO monitors (no longer manufactured) and Draeger and Gastec diffusion tubes have been used in field studies in the past. The Lascar monitors used in this study were electro-chemical like the HOBO monitors. It employed a sensor robust against co-pollutants, humidity, and high CO conditions. During our pilot studies, this instrument was validated against Gastec and Draeger diffusion tubes and found to have a high degree of agreement with Draeger tubes and a higher degree of agreement between co-located instruments compared with either Draeger or Gastec manufactured diffusion tubes. The use of continuous CO monitoring enabled us to observe diurnal and seasonal trends and thus distinguish contributions between heating and cooking.

The relative homogeneity of stoves and fuels amongst participating households in this study enabled us to link the diurnal variation in HAP with cooking vs. heating events, activity patterns, and personal exposures. Our study population used yak dung as the primary fuel and our findings were most comparable to households where people used biomass fuels, particularly dung. Therefore, our findings may be applicable to other cold or high-altitude regions, where local populations used biomass fuels and followed a pastoralist lifestyle.

## Conclusions

In this study, it was found that nearly ninety-nine percent of study participants had COHb that exceeded the WHO CO poisoning threshold in at least one of nine measurements. Cooking periods were found to have CO levels 7 times higher than overnight heating periods. The highest risk of acute CO poisoning during cooking events was observed among women and children, indicating the need for interventions targeting cooking exposures as a priority.

Overall, the findings emphasized the need for targeted interventions to reduce CO exposures during cooking and heating activities, with a particular focus on women and children, while also considering broader measures such as improved heating systems and smoking cessation programs.

## Figures and Tables

**Table 1:** Mean and standard deviation of COHb measured among males and females, stratified by activity.

Activity	Men who did not smoke during activity^a^ (measurements = 783)	Men who smoked during activity (measurements = 117)	Women^b^ (measurements = 900)
Resting	2.1 ± 1%	NA	1.8 ± 1%
Cooking	None	NA	4.2 ± 5.3%
Eating	2.6 ± 1.5%	NA	2.3 ± 1.1%
Other Indoor	2.4 ± 1.1%	NA	2 ± 1%
Outdoor	3.2 ± 1.6%^c^	4.4 ± 4.9%^c^	1.4 + 1.6%

a Smoking was classified based on whether participants had smoked in the four hours prior to measurement. Affirmative smoking reports exclusively occurred among men returning from herding.

b All women reported being non-smokers.

c Herding was the primary outdoor activity among men, taking ~12 h/day on average.

## Data Availability

The data used for analysis in this study is available from the authors upon request.

## References

[1] Smith KR, Bruce N, Balakrishnan K, Adair-Rohani H, Balmes J, Chafe Z (2014). Millions dead: how do we know and what does it mean? Methods used in the comparative risk assessment of household air pollution. Annual Review of Public Health.

[2] World Health Organization (2018). Burden of disease from household air pollution for 2016 Description of method.

[3] US EPA (2010). Integrated Science Assessment (ISA) for Carbon Monoxide (Final Report, Jan 2010), EPA/600/R-09/019F.

[4] Zhang J, Mauzerall DL, Zhu T, Liang S, Ezzati M, Remais JV (2010). Environmental health in China: progress towards clean air and safe water. Lancet.

[5] Zhang LX, Wang CB, Yang ZF, Chen B (2010). Carbon emissions from energy combustion in rural China. Procedia Environmental Sciences.

[6] Aunan K, Hansen MH, Liu Z, Wang S (2019). The hidden hazard of household air pollution in rural China. Environ Sci Policy.

[7] Lai AM, Carter E, Shan M, Ni K, Clark S, Ezzati M (2019). Chemical composition and source apportionment of ambient, household, and personal exposures to PM 2.5 in communities using biomass stoves in rural China. Sci Total Environ.

[8] Sinton JE, Smith KR, Peabody JW, Yaping L, Xiliang Z, Edwards R (2004). An assessment of programs to promote improved household stoves in China. Energy Sustain Dev.

[9] Qu J, Liu L, Zeng J, Maraseni TN, Zhang Z (2022). City-level determinants of household CO_2_ emissions per person: an empirical study based on a large survey in China. Land.

[10] Yu L, Ge Y, Tan J, He C, Wang X, Liu H (2014). Experimental investigation of the impact of biodiesel on the combustion and emission characteristics of a heavy duty diesel engine at various altitudes. Fuel.

[11] Kang S, Li C, Wang F, Zhang Q, Cong Z (2009). Total suspended particulate matter and toxic elements indoors during cooking with yak dung. Atmos Environ.

[12] Gao X, Yu Q, Gu Q, Chen Y, Ding K, Zhu J (2009). Indoor air pollution from solid biomass fuels combustion in rural agricultural area of Tibet, China. Indoor Air.

[13] Li C, Kang S, Chen P, Zhang Q, Guo J, Mi J (2012). Personal PM 2.5 and indoor CO in nomadic tents using open and chimney biomass stoves on the Tibetan Plateau. Atmos Environ.

[14] Carter E, Archer-Nicholls S, Ni K, Lai AM, Niu H, Secrest MH (2016). Seasonal and diurnal air pollution from residential cooking and space heating in the Eastern Tibetan Plateau. Environ Sci Technol.

[15] Ni K, Carter E, Schauer JJ, Ezzati M, Zhang Y, Niu H (2016). Seasonal variation in outdoor, indoor, and personal air pollution exposures of women using wood stoves in the Tibetan Plateau: baseline assessment for an energy intervention study. Environ Int.

[16] Ye W, Saikawa E, Avramov A, Cho SH, Chartier R (2020). Household air pollution and personal exposure from burning firewood and yak dung in summer in the eastern Tibetan Plateau. Environ Pollut.

[17] Sclar S, Saikawa E (2019). Household air pollution in a changing tibet: a mixed methods ethnography and indoor air quality measurements. Environ Manag.

[18] Cohen JE, Small C (1998). Hypsographic demography: the distribution of human population by altitude. Proc Natl Acad Sci USA.

[19] Beall CM, Laskowski D, Strohl KP, Soria R, Villena M, Vargas E (2001). Pulmonary nitric oxide in mountain dwellers. Nature.

[20] National Bureau of Statistics of China (2008). China Statistical Yearbook 2008.

[21] Fife CE, Koch S, Nguyen M, Otto GH, Wilhelm G (2001). A noninvasive method for rapid diagnosis of carbon monoxide poisoning.

[22] Jarvis MJ, Belcher M, Vesey C, Hutchison DC (1986). Low cost carbon monoxide monitors in smoking assessment. Thorax.

[23] Lam N, Nicas M, Ruiz-Mercado I, Thompson LM, Romero C, Smith KR (2011). Non-invasive measurement of carbon monoxide burden in Guatemalan children and adults following wood-fired temazcal (sauna-bath) use. J Environ Monit.

[24] Douglas CG, Haldane JS, Haldane JBS (1912). The laws of combination of hæmoglobin with carbon monoxide and oxygen. J Physiol.

[25] Wu L, Wang RUI (2005). Carbon monoxide: endogenous production. Physiol Funct Pharmacol.

[26] Raiyani CV, Shah SH, Desai NM, Venkaiah K, Patel JS, Parikh DJ (1993). Characterization and problems of indoor pollution due to cooking stove smoke. Atmos Environ Part A, Gen Top.

[27] Torres-Dosal A, Pérez-Maldonado IN, Jasso-Pineda Y, Martínez Salinas RI, Alegría-Torres JA, Díaz-Barriga F (2008). Indoor air pollution in a Mexican indigenous community: evaluation of risk reduction program using biomarkers of exposure and effect. Sci Total Environ.

[28] Zhou Z, Jin Y, Liu F, Cheng Y, Liu J, Kang J (2006). Community effectiveness of stove and health education interventions for reducing exposure to indoor air pollution from solid fuels in four Chinese provinces. Environ Res Lett.

[29] Denniston JC, Pettyjohn FS, Boyter JK, Kelliher JK, Hiott BF (1978). The interaction of carbon monoxide and altitude on aviator performance: pathophysiology of exposure to carbon monoxide. US Army Aeromed. Res Lab Rep.

[30] Lee A, Sanchez TR, Shahriar MH, Eunus M, Perzanowski M, Graziano J (2015). A cross-sectional study of exhaled carbon monoxide as a biomarker of recent household air pollution exposure. Environ Res.

[31] World Health Organization (1999). Environmental health criteria 213: carbon monoxide.

